# Allermatch™, a webtool for the prediction of potential allergenicity according to current FAO/WHO Codex alimentarius guidelines

**DOI:** 10.1186/1471-2105-5-133

**Published:** 2004-09-16

**Authors:** Mark WEJ Fiers, Gijs A Kleter, Herman Nijland, Ad ACM Peijnenburg, Jan Peter Nap, Roeland CHJ van Ham

**Affiliations:** 1 Applied Bioinformatics, Plant Research International, Wageningen University and Research Center, Wageningen, PO Box 16, 6700 AA, The Netherlands; 2 RIKILT-Institute of Food Safety, Wageningen University and Research Center, Wageningen, PO Box 230, 6700 AE, The Netherlands

## Abstract

**Background:**

Novel proteins entering the food chain, for example by genetic modification of plants, have to be tested for allergenicity. Allermatch™  is a webtool for the efficient and standardized prediction of potential allergenicity of proteins and peptides according to the current recommendations of the FAO/WHO Expert Consultation, as outlined in the Codex alimentarius.

**Description:**

A query amino acid sequence is compared with all known allergenic proteins retrieved from the protein databases using a sliding window approach. This identifies stretches of 80 amino acids with more than 35% similarity or small identical stretches of at least six amino acids. The outcome of the analysis is presented in a concise format. The predictive performance of the FAO/WHO criteria is evaluated by screening sets of allergens and non-allergens against the Allermatch databases. Besides correct predictions, both methods are shown to generate false positive and false negative hits and the outcomes should therefore be combined with other methods of allergenicity assessment, as advised by the FAO/WHO.

**Conclusions:**

Allermatch™ provides an accessible, efficient, and useful webtool for analysis of potential allergenicity of proteins introduced in genetically modified food prior to market release that complies with current FAO/WHO guidelines.

## Background

The safety of genetically engineered foods must be assessed before authorities in most nations will consider granting market approval. An important issue in current food safety assessment is the evaluation of the potential allergenicity of food derived from biotechnology. Since many food allergens are proteins, introduction of a new ("foreign") protein in food by genetic engineering can in theory cause allergic reactions. Therefore the allergenicity of novel proteins needs to be assessed. Potential allergenicity of a protein is a complex issue and various tests can be used for prediction, including bioinformatics, *in vitro *digestibility and binding of antisera of allergic patients. A step-by-step procedure to assess allergenicity is described by the Codex alimentarius and the FAO/WHO consultation group [1,2]. An important step in this procedure is to use bioinformatics to determine whether the primary structure (amino acid sequence) of a given transgenic protein is sufficiently similar to sequences of known allergenic proteins. The recommended procedure [1] to establish the possibility of allergenicity is to:

(1) Obtain the amino acids sequences of known allergens in protein databases in FASTA format (using the amino acids from the mature proteins only, disregarding the leader sequences, if any).

(2) Prepare the complete set of 80-amino acid length sequences derived from the query protein (again disregarding the leader sequence, if any).

(3) Compare each of the sequences of (2) with all sequences of (1), using the program FASTA [3] with default settings for gap penalty and extension.

According to the Codex alimentarius [2], potential allergenicity should be considered, when there is either:

(a) More than 35 % similarity over a window of 80 amino acids of the query protein with a known allergen.

(b) A stretch of identity of 6 to 8 contiguous amino acids.

This procedure is described in more detail by the expert consultation and the Codex Alimentarius. Potential allergenicity requires further testing of the protein with panels of patient sera and possibly animal exposure tests [1,2].

## Construction and content

**Figure 1:**
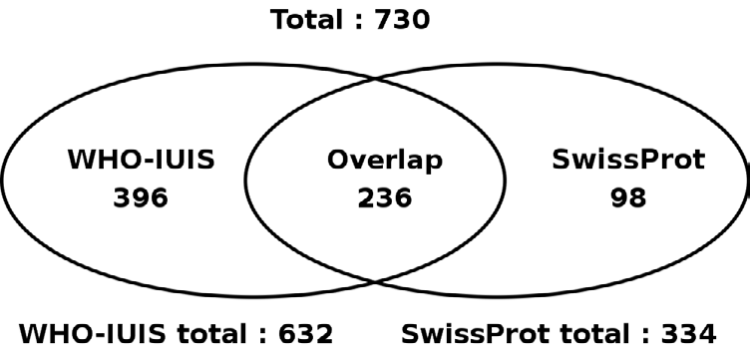
**A Venn-diagram showing the relationships of the three databases provided by Allermatch™. **This figure shows the size and overlap between the SwissProt and WHO-IUIS allergen databases.

Three allergen databases were created, one derived from SwissProt [4] and one from the WHO-IUIS allergen list [5]. A third database is a non-redundant combination of the other two. The databases were created by extracting all proteins from public databases; SwissProt (version 44.1, July 5 2004, [4]), PIR [6] and GenPept . Leader sequences were, if annotated, trimmed from the sequence. The SwissProt allergen list contains 334 mature protein sequences, while the WHO-IUIS allergen list (version June 7, 2004) contains 632 sequences (correcting for three internal duplications). These two databases contain 236 duplicate entries. The non-redundant combined database contains 730 sequences (Figure 1).

**Figure 2:**
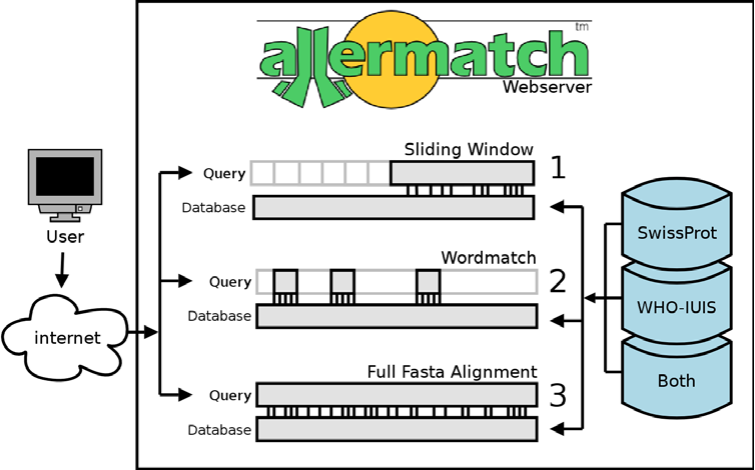
**Schematic representation of the Allermatch™ webtool. **The user submits a protein sequence of interest to the Allermatch™ webtool and chooses one of the three alignment methods and three databases available. Upon completion the results are formatted and returned to the user.

Allermatch™ is build around the FASTA package (version 3.4t21; , [3]) running with default parameters (ktup = 2, matrix = Blosum50, Gap open = -10, Gap extend = -2). The Allermatch™ analysis tool and the web interface are written in Python and run on a Suse L Linux Enterprise server with an Apache web server (version 1.3.26). Allermatch™ provides two search methods (mode 1 & 2) corresponding with the FAO/WHO guidelines described above and a third method (mode 3) is provided as an extra tool. The outline of the application is schematically presented in Figure 2.

### Mode 1: Sliding window approach

The query protein sequence is divided into 80 amino acid (aa) windows using a sliding window with steps of a single residue. Each of these windows is compared with all sequences in the allergen database of choice. All database entries showing a similarity higher than a configurable threshold percentage (default is 35%) to any of the 80 aa query sequence windows are flagged. Upon completion of the analysis, a table is shown with all flagged database entries. Per entry, the highest similarity score is given, as well as the number of windows having a similarity above the cut-off percentage. For each allergen database entry identified, more detailed information on the similarity between the allergen and query sequence can be retrieved, such as those areas of both proteins within all 80 aa windows scoring above the cut-off percentage. The similarity score calculated by FASTA can apply to stretches smaller than 80 aa, Allermatch™ converts such a similarity score to an 80 aa window. For example, 40% similarity on a stretch of 40 aa converts to 20% similarity on an 80 aa window.

### Mode 2: Wordmatch

This method looks for short sub-sequences (words), which have a perfect identity with a database entry. The wordsize is configurable (default is 6 aa). The output given is similar to the output given by Mode 1. All database entries with at least one hit are listed and for each of these, more detailed information can be retrieved upon request.

### Mode 3: full FASTA alignment with an Allermatch™ allergen database

The Allermatch™ webtool also offers a full alignment of the query sequence with either of the allergen databases using FASTA. Although this full alignment is currently not required by the FAO/WHO guidelines, the full alignment of protein sequences helps positioning of regions of potential allergenicity in the whole primary structure of the protein. The FASTA output is parsed and information from the allergen database is added and presented.

## Utility and discussion

To examine the predictive performance of the FAO/WHO criteria for potential allergenicity, we have performed two tests. The first test determines the percentage of false negative and the second test assesses the amount of false positives. Both tests are performed with standard settings; for the sliding window approach an 80 amino acid window with a 35% similarity cutoff is used and for the wordmatch approach 6, 7 and 8 aa word sizes are tested.

The false negative error-rate is estimated by a leave-one-out method, testing all sequences in each Allermatch™ database against that database with the tested sequence excluded. Each sequence not resulting in a hit is considered a false negative. The results of each method/database combination are summarized in Table 1, column 1. The results show that the number of false negatives decreases when a larger database of allergen sequences is used. This may (partly) be explained by an increased proportion of similar, but not equal, sequences in the larger databases, such as isoallergens listed by WHO-IUIS. In examining the results, various sequences were observed that were not able to produce a hit (data not shown) due to their short length, since a perfect hit on a sequence shorter than 28 amino acids cannot convert to a 35% hit on an 80 amino acid window. Column 2 of the same table shows the corrected false negative rate after exclusion of these sequences. Also after this correction the wordmatch with 6 amino acids method shows lower numbers of false negatives than the sliding window approach. It is clear, however, that in case of short protein sequences the sensitivity of the sliding window methods is reduced.

In the second test, we assess the odds of a false positive by testing 12 protein sequences known to be non allergenic. This is based on non-reactivity of these proteins towards IgE-sera of allergy patients or on the inability to cause IgE-responses in experimental animals (Table 2). It should be noted that such data are only available for a limited number of proteins, which accounts for the size of this dataset. Each of these 12 sequences was tested against all databases with all methods. Each non-allergenic sequence resulting in a hit is considered a false positive (Table 1, column 3). The number of false positives grows with the database size, as is to be expected: the chance of a random hit increases with a larger database. In contrast to the false negative hit rates the sliding window method gives the lower error rate. This test might, however, overestimate the number of false positives. A number of these non-allergens are related to and display similarities with their allergenic counterparts, *i.e. *T1 (related to Bet v 1), human serum albumin (related to animal serum albumins), and human heat shock protein 70 (similar to heat shock proteins from fungi and other allergens). A selection of unrelated, non-allergenic proteins is therefore likely to give a lower false positive rate. Caution should be taken in interpreting these false hit rates. The used methods might perform differently with other sets of proteins. For example, a member of a completely novel group of valid allergens is likely to generate a false negative result.

The imperfect results show here agree with literature where the FAO/WHO methods for sequence comparisons are also shown to lack full predictive capability [7-9]. Interestingly, the results show that there is a balance between false positives and negatives when increasing the threshold level for short exact matches from 6 to 8 amino acids, with the number of false positives sharply decreasing at 8 amino acids (Table 1). The outcomes of these tests therefore need to be further refined by checking for the presence of potential IgE-epitopes as recommended by Kleter and Peijnenburg [7], as well as combined with results of other assays as recommended by the Codex. Other methods to decrease false hit rates may also be considered [8,9]. We plan to implement such supplementary methods in the future to support the Codex based predictions of potential allergenicity.

The prediction of potential allergenicity by primary sequence comparison depends on the quality of the data used for comparison. Addition of a non-allergenic or poorly annotated protein to any of the Allermatch™ allergen databases would obviously result in undesired false positives and should be prevented. A workable strategy could be to use multiple databases, *i.e. *a database based on SwissProt's list of allergens, which contains well-annotated sequences from SwissProt, simultaneously with a larger database based on the WHO-IUIS list, which contains possibly less well annotated sequences from other protein databases, such as GenPept. For example, a number of protein accessions in the WHO-IUIS database do not mention the presence of signal- and/or pro-peptides, where removal of such peptides is essential to prevent false positives. Users of Allermatch™ should, at all times, take into account the possibility of a false positive or negative, for example by checking original data (accessions, clinical literature) and confirm results, before arriving at conclusions. To prevent false positives as much as possible, one should choose for the well-annotated SwissProt database. To prevent false negatives, the combination of the larger WHO-IUIS database with that of SwissProt is more appropriate. Updates to the SwissProt and WHO-IUIS allergen lists will be incorporated in the Allermatch™ databases on a regular basis.

Several other websites in the public domain offer sequence alignment facilities that support the prediction of potential allergenicity, such as SDAP [10,11], AllerPredict [12] and Farrp [13]. These websites offer search algorithms that find contiguous similar amino acids between a query sequence and database sequences (SDAP, AllerPredict) and more than 35% identity in alignments (SDAP, AllerPredict), as well as a general FASTA of a query protein sequence against the database (SDAP, Farrp).

## Conclusions

Allermatch™ is an efficient and comprehensive webtool that combines all bioinformatics approaches required to assess the allergenicity of protein sequences according to the current guidelines in the Codex. The application will be kept up to date with the FAO/WHO criteria and the SwissProt and WHO-IUIS allergen lists. It will be extended with other, supplementary methods to support and refine the prediction of allergenicity.

## Availability and requirements

Allermatch™ is platform independent and accessible using any Netscape 4+ compatible webbrowser at .

## Authors' contributions

MF developed and implemented the Allermatch™ webtool. HN provided the domain name registration and advised in the web site development. GK and AP provided the scientific background and constructed the sequence databases. JPN and RvH provided time, resources and ample discussion. All authors have read and approved the final manuscript.

## Figures and Tables

**Table 1:** Prediction quality of the FAO/WHO methods.

			1		2		3	
			
			False negatives		False negatives (corrected)		False positives	
			
Database	Method	Wordsize	Number	%	Number	%	Number	%
SwissProt	Window	n.a.	71 / 334	21.3	57 / 320	17.8	3 / 12	25.0
	Wordmatch	6	54 / 334	16.2	n.a.	n.a.	7 / 12	58.3
		7	69 / 334	20.7	n.a.	n.a.	6 / 12	50.0
		8	78 / 334	23.4	n.a.	n.a.	3 / 12	25.0
WHO-IUIS	Window	n.a.	99 / 632	15.7	78 / 611	12.8	4 / 12	33.3
	Wordmatch	6	58 / 632	9.2	n.a.	n.a.	9 / 12	75.0
		7	98 / 632	15.5	n.a.	n.a.	8 / 12	66.7
		8	117 / 632	18.5	n.a.	n.a.	3 / 12	25.0
SwissProt & WHO-IUIS	Window	n.a.	101 / 730	13.8	77 / 706	10.9	5 / 12	41.7
	Wordmatch	6	55 / 730	7.5	n.a.	n.a.	9 / 12	75.0
		7	95 / 730	13.0	n.a.	n.a.	8 / 12	66.7
		8	115 / 730	15.8	n.a.	n.a.	3 / 12	25.0

The number and percentage of false negative and false positive results are shown here for all FAO/WHO recommended method/database combinations. Result set 1 describes the number of false negatives observed in a leave-one-out approach. The next result set (2) shows the same results but corrected for those sequences that were not able to generate a hit against itself due to the short length of the sequence. The last result set (3) shows the observed number of false positives when testing 12 non-allergenic sequences with the Allermatch™ webtool. Each of the result sets consists of two columns; the first column shows the number of erroneous hits and the total number of sequences in this set. The second column shows the percentage of erroneous hits.

**Table 2:** Sequences used for the negative control

Protein	Host organism	Evidence for non-allergenicity	Accession	Reference
Amaranth seed albumin	*Amaranthus hypochondriacus*	IgG-response, but no raised IgE-levels, after administration (intranasal and intraperitoneal) of amaranth seed albumin to mice	GenPept CAA77664	[14]
T1	*Catharanthus roseus*	No reaction of recombinant T1 in IgE-sera binding, basophile histamine release, and skin prick testing using patients allergic to the related birch pollen allergen Bet v 1	Not applicable	[15]
Mite ferritin heavy chain	*Dermatophagoides pteronyssinus*	Reaction of mite ferritin with IgG, but not with IgE, of sera from patients allergic to house dust mite	GenPept AAG02250	[16]
Maltose binding protein	*Escherichia coli*	No reaction with IgE-sera from patients allergic to natural rubber latex (maltose binding protein used as part of fusion proteins with latex allergens)	SwissProt P02928	[17]
Human serum albumin	*Homo sapiens*	No reaction of human serum albumin with IgE-sera of patients allergic to cat- and porcine-serum albumin	SwissProt P02768	[18]
Human heat shock protein 70	*Homo sapiens*	No reaction of human heat shock protein 70 with IgE-sera of patients allergic to heat shock protein 70 from *Echinococcus granulosus*	SwissProt P08107	[19]
Human beta-2-glycoprotein I	*Homo sapiens*	Presence of IgM antibodies, but not of IgE antibodies, directed against human beta-2-glycoprotein I in sera from atopic eczema/dermatitis patients	SwissProt P02749	[20]
Guayule rubber particle protein	*Parthenium argentatum*	No cross-reactivity between proteins from guayule and latex using IgE-sera from patients allergic to latex	Swissprot Q40778	[21]
Purple acid phosphatase 1	*Solanum tuberosum*	Stimulation of IgG-, but no or only low stimulation of IgE-antibodies following administration of potato acid phosphatase to mice (oral and intraperitoneal)	TrEMBL Q6J5M7	[22]
Purple acid phosphatase 2	*Solanum tuberosum*	See above	TrEMBL Q6J5M9	[22]
Purple acid phosphatase 3	*Solanum tuberosum*	See above	TrEMBL Q6J5M8	[22]
Potato lectin	*Solanum tuberosum*	Stimulation of IgG-, but no or only low stimulation of IgE-antibodies following administration of potato lectin to mice (intraperitoneal)	TrEMBL Q9S8M0	[23]
